# Estimated economic impact of vaccinations in 73 low- and middle-income countries, 2001–2020

**DOI:** 10.2471/BLT.16.178475

**Published:** 2017-06-27

**Authors:** Sachiko Ozawa, Samantha Clark, Allison Portnoy, Simrun Grewal, Meghan L Stack, Anushua Sinha, Andrew Mirelman, Heather Franklin, Ingrid K Friberg, Yvonne Tam, Neff Walker, Andrew Clark, Matthew Ferrari, Chutima Suraratdecha, Steven Sweet, Sue J Goldie, Tini Garske, Michelle Li, Peter M Hansen, Hope L Johnson, Damian Walker

**Affiliations:** a Division of Practice Advancement and Clinical Education, UNC Eshelman School of Pharmacy, University of North Carolina at Chapel Hill, CB # 7574, Beard Hall 115H, Chapel Hill, North Carolina, 27599, United States of America (USA).; b Department of International Health, Johns Hopkins Bloomberg School of Public Health, Baltimore, USA.; c Department of Global Health and Population, Harvard TH Chan School of Public Health, Boston, USA.; d Department of Pharmacy, University of Washington, Seattle, USA.; e Independent Consultant, Denver, USA.; f Department of Preventive Medicine and Community Health, Rutgers New Jersey Medical School, Newark, USA.; g Centre for Health Economics, University of York, York, England.; h Department of Health Services, Norwegian Institute of Public Health, Oslo, Norway.; i Department of Health Services Research and Policy, London School of Hygiene & Tropical Medicine, London, England.; j Center for Infectious Disease Dynamics, The Pennsylvania State University, University Park, USA.; k Independent Consultant, Vienna, USA.; l Center for Health Decision Science, Harvard TH Chan School of Public Health, Boston, USA.; m MRC Centre for Outbreak Analysis and Modelling, Imperial College London, London, England.; n Gavi, the Vaccine Alliance, Geneva, Switzerland.; o The Global Fund to Fight AIDS, Tuberculosis and Malaria, Geneva, Switzerland.; p Bill & Melinda Gates Foundation, Seattle, USA.

## Abstract

**Objective:**

To estimate the economic impact likely to be achieved by efforts to vaccinate against 10 vaccine-preventable diseases between 2001 and 2020 in 73 low- and middle-income countries largely supported by Gavi, the Vaccine Alliance.

**Methods:**

We used health impact models to estimate the economic impact of achieving forecasted coverages for vaccination against *Haemophilus influenzae* type b, hepatitis B, human papillomavirus, Japanese encephalitis, measles, *Neisseria meningitidis* serogroup A, rotavirus, rubella, *Streptococcus pneumoniae* and yellow fever. In comparison with no vaccination, we modelled the costs – expressed in 2010 United States dollars (US$) – of averted treatment, transportation costs, productivity losses of caregivers and productivity losses due to disability and death. We used the value-of-a-life-year method to estimate the broader economic and social value of living longer, in better health, as a result of immunization.

**Findings:**

We estimated that, in the 73 countries, vaccinations given between 2001 and 2020 will avert over 20 million deaths and save US$ 350 billion in cost of illness. The deaths and disability prevented by vaccinations given during the two decades will result in estimated lifelong productivity gains totalling US$ 330 billion and US$ 9 billion, respectively. Over the lifetimes of the vaccinated cohorts, the same vaccinations will save an estimated US$ 5 billion in treatment costs. The broader economic and social value of these vaccinations is estimated at US$ 820 billion.

**Conclusion:**

By preventing significant costs and potentially increasing economic productivity among some of the world’s poorest countries, the impact of immunization goes well beyond health.

## Résumé

**Objectif:**

Estimer l'impact économique qui pourrait découler des efforts de vaccination contre 10 maladies à prévention vaccinale déployés entre 2001 et 2020 dans 73 pays à revenu faible et intermédiaire, et largement soutenus par Gavi, l'Alliance du Vaccin.

**Méthodes:**

Nous avons utilisé des modèles d'évaluation de l'impact sur la santé pour estimer l'impact économique qui découlerait, si le taux de couverture prévu est atteint, des vaccinations contre *Haemophilus influenzae* type B, l'hépatite B, le papillomavirus humain, l'encéphalite japonaise, la rougeole, *Neisseria meningitidis* sérogroupe A, le rotavirus, la rubéole, *Streptococcus pneumoniae* et la fièvre jaune. Pour établir une comparaison avec l'absence de vaccination, nous avons modélisé les coûts – exprimés en dollars des États-Unis 2010 (USD) – des traitements évités, les coûts de transport, les pertes de productivité des soignants non professionnels et les pertes de productivité pour cause d'invalidité ou de décès. Nous avons utilisé une méthode permettant d'évaluer la valeur d'une année de vie pour estimer la valeur économique et sociale au sens large d'une vie plus longue et en meilleure santé grâce à la vaccination.

**Résultats:**

D'après nos estimations, les vaccinations pratiquées entre 2001 et 2020 dans les 73 pays permettront d'éviter plus de 20 millions de décès et d'économiser 350 milliards de dollars des États-Unis en coûts sanitaires. Les cas de décès et d'invalidité évités grâce à la vaccination pratiquée au cours de ces deux décennies entraîneront des gains de productivité permanents respectivement estimés à 330 milliards de dollars des États-Unis et 9 milliards de dollars des États-Unis. On estime qu'au cours de la vie des cohortes vaccinées, les mêmes vaccinations permettront d'économiser 5 milliards de dollars des États-Unis en coûts de traitement. La valeur économique et sociale au sens large de ces vaccinations est estimée à 820 milliards de dollars des États-Unis.

**Conclusion:**

L'impact de la vaccination dépasse le domaine de la santé, car il permet d'éviter d'importants coûts et une augmentation potentielle de la productivité économique de certains des pays les plus pauvres du monde.

## Resumen

**Objetivo:**

Estimar el impacto económico que probablemente se logaría con los esfuerzos de vacunar frente a 10 enfermedades evitables mediante la vacunación entre 2001 y 2020 en 73 países con ingresos bajos y medios ampliamente respaldados por la Gavi, la Vaccine Alliance.

**Métodos:**

Se utilizaron modelos de impacto sanitario para estimar el impacto económico de lograr las coberturas previstas de vacunación frente a *Haemophilus influenzae* tipo b, hepatitis B, virus del papiloma humano, encefalitis japonesa, sarampión, *Neisseria meningitidis* serogrupo A, rotavirus, rubéola, *Streptococcus pneumoniae* y fiebre amarilla. En comparación con la no vacunación, se modelaron los costes (expresados en dólares estadounidenses, USD, de 2010) de los tratamientos evitados, los costes de transporte, las pérdidas de productividad de los proveedores de salud y las pérdidas de productividad debido a la discapacidad y la muerte. Se utilizó el método de valor de vida anual para estimar de forma más amplia el valor económico y social del hecho de vivir más, con una mejor salud, como resultado de la inmunización.

**Resultados:**

Se estimó que, en los 73 países, las vacunas suministradas entre 2001 y 2020 evitarán más de 20 millones de muertes y ahorrarán 350 000 millones de USD en costes de enfermedades. Las muertes y las discapacidades evitadas gracias a las vacunas suministradas durante las dos décadas tendrán como resultado unas ganancias permanentes estimadas en la productividad de un total de 330 000 millones de USD y 9 000 millones de USD, respectivamente. Durante la vida de las cohortes vacunadas, se estima que las mismas vacunaciones ahorrarán 5 000 millones de USD en costes de tratamientos. El valor económico y social más amplio de estas vacunas se estima en 820 000 millones de USD.

**Conclusión:**

El impacto de las vacunas es positivo más allá de la salud, ya que se evitan costes significativos y se aumenta potencialmente la productividad económica entre algunos de los países más pobres.

## ملخص

الغرض ****تقدير الأثر الاقتصادي المحتمل من خلال الجهود المبذولة للتمنيع ضد 10 أمراض يمكن الوقاية منها باللقاحات في الفترة ما بين عامي 2001 و2020 في 73 بلدًا من البلدان منخفضة ومتوسطة الدخل التي تتلقى دعمًا واسع النطاق من جانب التحالف العالمي للقاحات والتحصين (Gavi) لإنتاج الأمصال.

الطريقة ****استخدمنا نماذج الأثر الصحي لتقدير الأثر الاقتصادي لتحقيق التغطية المتوقعة للتمنيع ضد المستدمية النزلية من النوع (ب)، وفيروس التهاب الكبد (ب)، وفيروس الورم الحليمي البشري، والتهاب الدماغ الياباني، والحصبة، والزمرة المصلية النيسرية السحائية (أ)، والفيروس العجلي، والحصبة الألمانية، والعقدية الرئوية، والحمى الصفراء. وبالمقارنة مع عدم التمنيع، قمنا بوضع نموذج للتكاليف – المحتسبة في عام 2010 بالدولار الأمريكي – للمعالجة التي تم تفاديها وتكاليف النقل وخسائر الإنتاجية لدى مقدمي الرعاية وخسائر الإنتاجية بسبب العجز والوفاة. وقد استخدمنا طريقة التقييم على أساس العمر لتقدير القيمة الاقتصادية والاجتماعية الأوسع للعيش لفترة أطول، في صحة أفضل، نتيجة للحصول على التمنيع.

النتائج تشير تقديراتنا إلى أن اللقاحات الممنوحة في الفترة ما بين عامي 2001 و2020 ستؤدي في 73 بلدًا إلى تفادي أكثر من 20 مليون حالة وفاة وتوفير 350 مليار دولار من تكاليف المرض. وستؤدي حالات الوفيات والعجز التي تم تمنيعها باللقاحات خلال العقدين إلى تحقيق مكاسب إنتاجية تستمر مدى الحياة بإجمالي 330 مليار دولار أمريكي و9 مليارات دولار أمريكي على التوالي. وعلى مدى عمر المجموعات الملقحة، فإن نفس عمليات التمنيع ستوفر ما يقدر بـ 5 مليارات دولار في تكاليف العلاج. وتُقدر القيمة الاقتصادية والاجتماعية الأوسع لهذه اللقاحات بمبلغ 820 مليار دولار أمريكي.

الاستنتاج من خلال منع التكاليف الباهظة واحتمال زيادة الإنتاجية الاقتصادية بين بعض أفقر بلدان العالم، فإن أثر التمنيع يتخطى مجرد تحقيق منافع صحية.

## 摘要

**目的:**

评估 2001 到 2020 年间 73 个中低收入国家通过针对 10 种疫苗可预防疾病进行疫苗接种可能实现的经济影响。评估主要由全球疫苗联盟 (Gavi) 提供支持。

**方法:**

我们使用健康影响模型评估了实现预期的*乙型流感嗜血杆菌*、乙型肝炎、人乳头瘤病毒、乙型脑炎、麻疹、A 群*脑膜炎奈瑟菌*、轮状病毒、风疹、*肺炎双球菌*和黄热病疫苗接种覆盖率的经济影响。 与不进行疫苗接种相比,我们以 2010 年的美元 (US$) 为单位建立了成本模型——包括避免治疗节省的成本、运输成本、护理人员的生产力损失和因残疾和死亡造成的生产力损失。 我们使用“年生命值”方法估算了因免疫接种而延长生命和保持更健康的生活的更广泛经济和社会价值。

**结果:**

我们估计 2001 年到 2020 年间,73 个国家的疫苗接种将避免超过 2000 万人的死亡数和节省 3500 亿美元的疾病成本。 二十年间通过疫苗接种避免的死亡和残疾数预计将分别带来总额为 3300 亿美元和 90 亿美元的终身生产力收益。 在接种疫苗人群的一生中接种同种疫苗预计会节省 50 亿的治疗成本。 预计此类疫苗接种的更广泛经济和社会价值达 8200 亿美元。

**结论:**

通过减少世界上一些最贫穷国家的重大开支和提高潜在的经济生产力,免疫接种的影响远不止在卫生领域。

## Резюме

**Цель:**

Оценить экономические последствия, которые могут быть достигнуты благодаря усилиям в области вакцинации против 10 предупреждаемых вакцинацией болезней, в период с 2001 по 2020 г. в 73 странах с низким и средним уровнем дохода, которые в значительной степени поддерживаются ГАВИ (Глобальным альянсом по вакцинам и иммунизации).

**Методы:**

Мы использовали модели воздействия на здоровье для оценки экономических последствий достижения прогнозируемых покрытий вакцинацией против таких возбудителей инфекций, как Haemophilus influenzae типа b, вирус гепатита B, вирус папилломы человека, вирус японского энцефалита, вирус кори, Neisseria meningitidis серогруппы A, ротавирус, вирус краснухи, Streptococcus pneumoniae и вирус желтой лихорадки. При сравнении с отсутствием вакцинации мы моделировали затраты, выраженные в долларах США (US $) по курсу 2010 года: предотвращение расходов на лечение, транспортных расходов, нетрудоспособности лиц, ухаживающих за детьми, и нетрудоспособности из-за инвалидности и смерти. Мы использовали метод, основанный на ценности одного года жизни, чтобы оценить более широкую экономическую и социальную ценность более долгой жизни и лучшего состояния здоровья, достигнутых в результате иммунизации.

**Результаты:**

Мы подсчитали, что в 73 странах вакцинации, проведенные в период с 2001 по 2020 г., предотвратят более 20 миллионов смертей и позволят сэкономить 350 миллиардов долларов США на затратах, связанных с болезнями. Смертность и инвалидность, предотвращенные с помощью вакцинации, проведенной в течение двух десятилетий, приведут к ожидаемому росту производительности труда на протяжении всей жизни на общую сумму 330 миллиардов долларов США и 9 миллиардов долларов США соответственно. В течение жизни вакцинированных когорт те же вакцинации позволят сэкономить приблизительно 5 миллиардов долларов США на расходах на лечение. Более широкая экономическая и социальная ценность этих вакцинаций оценивается в 820 миллиардов долларов США.

**Вывод:**

Предотвращая значительные издержки и потенциально увеличивая экономическую производительность среди некоторых беднейших стран мира, влияние иммунизации выходит далеко за пределы здоровья.

## Introduction

While vaccination is generally regarded to be one of the most cost-effective interventions in public health, the introduction and sustained use of any new vaccine needs to be supported by decision-makers who appreciate the full potential economic benefits that result.^1^^–^^5^ This paper focuses on the economic benefits of the vaccinations given, against 10 diseases, in 73 low- and middle-income countries supported by Gavi, the Vaccine Alliance, since Gavi’s establishment in 2001.

In 2011, disease modelling experts were convened, by Gavi and the Bill & Melinda Gates Foundation, to estimate the global impact of immunization beyond the original Expanded Programme on Immunization – based on the latest forecasts of vaccine demand and estimates of disease burden. In 2013, these experts developed health impact models to estimate the numbers of cases of illness, deaths and disability-adjusted life-years (DALYs) averted as the result of vaccination against 10 diseases in 73 low- or middle-income countries.^6^^,^^7^ Recently, we built on the output from these models by estimating the corresponding economic impact. To reflect the full impact of vaccinations in low-and middle-income countries, we captured not only the traditional costs of illness – e.g. productivity losses averted and treatment costs saved – but also projected the long-term economic and social benefits of vaccinations.^2^

## Methods

### Cost of illness

Since 2001, 73 countries with per-capita gross national incomes in 2003 of no more than 1000 United States dollars (US$) have received Gavi support. Our analysis was based on data for these 73 low- or middle-income countries (Box 1). We estimated the health and economic impact of vaccination against 10 vaccine-preventable diseases: *Haemophilus influenzae* type b, hepatitis B, human papillomavirus, Japanese encephalitis, measles, *Neisseria meningitidis* serogroup A, rotavirus, rubella, *Streptococcus pneumoniae* and yellow fever. Table 1 presents the relevant health outcomes, permanent disabilities and vaccines that we included in our models. While all of our study vaccines are delivered via routine immunization, supplementary immunization activities also occur for Japanese encephalitis, measles, *N. meningitidis* serogroup A, rubella and yellow fever. Our estimates of the numbers of deaths, cases and DALYs averted as the result of vaccination were developed from health impact models, as previously described.^6^^,^^7^ We used data on immunization coverages published by the World Health Organization (WHO) and United Nations Children’s Fund to estimate annual vaccine coverages for each study country for the period 2001–2012^8^ and version 9 of Gavi’s strategic demand forecast to estimate the corresponding probable coverages for the period 2013–2020.^9^

Box 1 **Countries included in the analysis on the estimated economic impact of vaccinations, 2001–2020**

Afghanistan, Angola, Armenia, Azerbaijan, Bangladesh, Benin, Bhutan, Bolivia (Plurinational State of), Burkina Faso, Burundi, Cambodia, Cameroon, Central African Republic, Chad, Comoros, Congo, Côte d'Ivoire, Cuba, Democratic People's Republic of Korea, Democratic Republic of the Congo, Djibouti, Eritrea, Ethiopia, Gambia, Georgia, Ghana, Guinea, Guinea-Bissau, Guyana, Haiti, Honduras, India, Indonesia, Kenya, Kiribati, Kyrgyzstan, Lao People's Democratic Republic, Lesotho, Liberia, Madagascar, Malawi, Mali, Mauritania, Mongolia, Mozambique, Myanmar, Nepal, Nicaragua, Niger, Nigeria, Pakistan, Papua New Guinea, Republic of Moldova, Rwanda, Sao Tome and Principe, Senegal, Sierra Leone, Solomon Islands, Somalia, South Sudan, Sri Lanka, Sudan, Tajikistan, Timor-Leste, Togo, Uganda, Ukraine, United Republic of Tanzania, Uzbekistan, Viet Nam, Yemen, Zambia and Zimbabwe.

**Table 1:** Model parameters for 10 vaccine-preventable diseases

Pathogen	Vaccines	Target disease	Death and disabilities adverted
*Haemophilus influenzae* type b	Pentavalent	Meningitis and pneumonia	Death, deafness, cognitive impairment, motor impairment and seizure disorder
Hepatitis B	Monovalent, tetravalent (DTP–HepB) or pentavalent (DTP–HepB–Hib)	Acute/fulminant infection, chronic infection, hepatocellular carcinoma, compensated and decompensated cirrhosis	Death
Human papillomavirus	Recombinant quadrivalent or bivalent	Cervical cancer	Death
Japanese encephalitis	Live attenuated	Japanese encephalitis	Death, neurological sequelae/cognitive impairment
Measles^a^	Live attenuated (measles or measles–rubella)	Measles	Death, central nervous system sequelae
*Neisseria meningitidis* serogroup A	Conjugate	Meningitis	Death, deafness, vision impairment, motor impairment and seizure disorder
Rotavirus	Attenuated oral rotavirus (RV1 or RV5)	Severe and non-severe diarrhoea	Death
Rubella	Live attenuated (rubella or measles–rubella)	Congenital rubella syndrome	Death, hearing loss, vision loss, cardiac abnormalities and central nervous system complications
*Streptococcus pneumoniae*	Conjugate (PCV10 or PCV13)	Meningitis and pneumonia	Death, deafness, cognitive impairment, motor impairment and seizure disorder
Yellow fever	Live attenuated (17D)	Yellow fever disease	Death

DTP: diphtheria–tetanus–pertussis; HepB: hepatitis B; Hib: *Haemophilus influenzae* type b; PCV10: 10-valent pneumococcal conjugate vaccine; PCV13: 13-valent pneumococcal conjugate vaccine; RV1: Rotarix®; RV5: RotaTeq®.

^a^ For our analysis, we only captured the benefits of second doses of measles vaccine and measles-related supplementary immunization activities. We did not estimate the impact of the first doses as they formed a standard component of national immunization programmes.

Using the cost of illness approach^10^ from a societal perspective, we estimated treatment costs and productivity losses averted by vaccination based on the estimated numbers of cases, deaths and disabilities averted.^11^ For each of the 10 diseases studied, we constructed decision tree models to capture both the short- and long-term averted costs of illness. These costs were broken down into five categories: (i) averted treatment costs; (ii) averted transportation costs for seeking care; (iii) averted reduction in caregivers’ economic output; (iv) averted loss of productivity due to premature death; and (v) averted loss of survivors’ productivity due to disability. All estimates of the averted cost of illness were discounted at 3% and are expressed in 2010 US$. We present separate results for the 20 years following Gavi’s establishment – i.e. 2001–2020 – and the current so-called Decade of Vaccines – i.e. 2011–2020.

In estimating the immunization-attributable averted costs of treatment, transportation and lost caregiver productivity, we used the country-specific estimated proportions of children for whom care was sought^12^ as well as data on the duration and rates of hospital admission.^13^^–^^18^ Country-specific costs of relevant inpatient and outpatient care at hospitals and health centres were primarily obtained from the WHO’s Choosing Interventions that are Cost-Effective (WHO-CHOICE) project.^19^ Costs of medications and diagnostics were estimated as proportions of facility costs. We assumed that each inpatient admission or outpatient visit was associated with a fixed transportation cost – i.e. a country-specific estimated mean cost of a return trip to and from a health-care facility.^20^ We also assumed that caregivers of sick children lost half their daily productivity for an outpatient visit and a full day’s productivity for each day a child was hospitalized. In each study country, a caregiver’s daily productivity was assumed to equal the daily minimum wage.^21^

Lost productivity resulting from convalescence and long-term disability was estimated for cases that could be averted by vaccination. To account for other causes of mortality that may impact the number of survivors entering the workforce, age-specific survival rates were applied to the non-fatal cases. The total number of productive years lost due to disability was estimated using the difference between life expectancy and mean age at disability onset – incorporating relevant disability weights. Estimates of life expectancy were derived from data published by the United Nations’ Population Division^22^ and disability weights from the 2010 Global Burden of Disease study.^23^ For each study country, we estimated lost productivity resulting from disability and premature mortality by multiplying the number of productive life-years lost due to disability or premature death by the projected annual values for the per-capita gross domestic product.^24^ The values we give for total averted long-term productivity losses represent the projected economic outputs of children whose disability or death are – or will be – prevented through immunization. Children were assumed to begin their economically productive lives when they reached an age of 15 years. Further detail on the key inputs, assumptions and data sources used for our analysis has been published.^7^

### Economic and social value

We used a second method to capture the broader economic and social value placed on living longer and healthier lives as a result of vaccination. For this, we applied a value-of-life approach that provides a societal perspective of the full benefits of reduced mortality. The estimated value of a life-year was based on data from two sources: (i) wage risk studies that use data on labour markets to examine the trade-off between wages and risk of mortality while employed; and (ii) stated preference studies in which individuals are asked how much they are willing to pay to avoid certain risks of death.^25^^,^^26^ Based on earlier work to estimate the annual per-capita value of an increase in life expectancy,^27^^–^^30^ we assumed that the value of a life-year saved in a particular country was 1.6 times that country’s annual per-capita gross domestic product. The economic and social value of vaccinations was estimated from the number of deaths averted due to vaccines, the difference between life expectancy and mean age of death from each study disease and the relevant per-capita gross domestic product (GDP).^31^

Traditional estimates of the value of a life-year have focused on estimating the full benefits of mortality reduction – with few studies examining the impact of corresponding reductions in morbidity.^28^^,^^32^ To reflect the benefit of averting morbidity, we estimated the value of a year lived with disability. As in the estimation of years lived with disability and years of life lost – both used to calculate a DALY – disability weights were applied to estimate the impact of various disabling conditions on an individual’s value of life from the age of disease onset to expected age at death.^22^ Disability weights varied from 0 – representing perfect health – to 1 – representing death. A similar approach was used in a previous estimation of the impact of disability on the value of a life-year.^28^ Our estimate of the value of a year lived with disability was used to estimate the full economic loss associated with permanent, long-term disability caused by any of the six study diseases that can have permanent sequelae – i.e. *H. influenzae* type b, Japanese encephalitis, measles, *N. meningitidis* serogroup A, rubella and *S. pneumoniae*.

### Sensitivity analysis

Multivariate Monte Carlo simulations, with 10 000 replications, were performed to assess the impact on cost estimates of uncertainty in the values of several key parameters: labour-force participation, per-capita GDP, the numbers of cases and deaths averted, the multiplier – otherwise set at 1.6 – used in the estimation of the value of a life-year, transportation costs and WHO-CHOICE treatment costs. Cost values were sampled from *γ* distributions to represent the right skew of observed costing data while non-cost values were sampled from *β* distributions. Lower and upper ranges of distributions were derived from health impact models or published literature. Results from the sensitivity analysis were used to construct 90% uncertainty ranges around point estimates and generate a tornado diagram illustrating the degree to which individual parameters influenced the final results. The analysis was performed using version 6 of the @RISK software package (Palisade Corporation, Ithaca, United States of America).

## Results

### Cost of illness

Table 2 presents estimates of the health and economic impact, for the period of 2001–2020 and the decade of 2011–2020, of averting 10 vaccine-preventable diseases in the 73 countries. According to our analyses, use of life-saving vaccines will avert an estimated 20 million deaths, 500 million cases of illness, 9 million cases of long-term disability and 960 million DALYs between 2001 and 2020. During the Decade of Vaccines, introduction and/or increased coverage of the modelled vaccines are projected to avert over 14 million deaths, 350 million cases of illness, 8 million cases of long-term disability and 700 million DALYs.

**Table 2:** Estimated economic and health benefits of vaccinations against 10 diseases, 73 Gavi-supported low- and middle-income countries, 2001–2020 and 2011–2020

Period, pathogen	Mortality and morbidity averted^a^				DALYs averted (millions)^a^	Averted costs of illness^b^						Value of life-year of disability averted (billions of US$)^b^	Economic and social value (billions of US$)^b^
	Deaths (thousands)	Cases (millions)	Long-term disability (thousands)^c^	Acute disease hospitalizations (millions)		Treatment costs (millions of US$)	Transport costs (millions of US$)	Lost caregiver wages (millions of US$)	Productivity loss due to disability (billions of US$)^c^	Productivity loss due to death (billions of US$)	Total (billions of US$)		
**2001–2020**													
HepB	7 200	120	N/A	0.5	210	1 148.8	134.4	25.2	1.3	46.9	49.5	N/A	81.3
Hib	2 700	83	4 900	25	180	1 815.8	94.6	399.9	2.5	58.9	63.7	7.4	180.1
HPV	850	1.1	N/A	1.1	16	6	0.9	N/A	0.9	3.2	4	NA	5
JE	59	0.3	67	0.2	8.4	33.5	0.2	1.6	0.4	2.1	2.5	0.6	4.2
Measles	5 100	210	350	21	310	361.7	168.6	223.7	1.4	139.8	142	3.6	349.4
NmA	470	3.1	380	1.4	23	39.8	1.2	0.2	0.7	7.8	8.6	1.1	13.7
Rotavirus	390	21	N/A	0.7	25	45	17.6	22.8	N/A	8.7	8.8	N/A	25.8
Rubella	280	0.9	270	0.5	26	19	1.3	16	0.6	4.5	5.1	1.5	14.2
Sp	1 700	51	3 200	14	110	773.9	51.3	197.2	1.5	31	33.5	4.3	96.2
YF	1 600	7.8	N/A	5.3	57	336.7	20.6	N/A	0.2	28.9	29.4	N/A	46.2
Total	20 000	500	9 200	70	960	4 580.2	490.8	886.4	9.4	331.8	347.1	18.6	816.1
**2011–2020**													
HepB	4 700	80	N/A	0.3	140	719.2	87	16.1	0.8	28.8	30.4	N/A	50.1
Hib	2 200	72	4 300	22	150	1 542	82.7	347.5	2.2	49.4	53.6	6.4	150.9
HPV	850	1.1	NA	1.1	16	6	1.2	NA	0.9	3.2	4	N/A	5
JE	38	0.2	44	0.1	6.9	21.3	0.2	1.5	0.2	1.3	1.5	0.4	2.7
Measles	2 900	120	200	13	180	223.1	96.7	131.2	0.9	87.1	88.4	2.2	218.2
NmA	440	3	350	1.3	21	38.4	1.2	0.2	0.7	7.5	8.3	1.1	13.1
Rotavirus	390	21	N/A	0.7	25	44.7	17.5	22.6	N/A	8.7	8.8	N/A	25.7
Rubella	260	0.9	250	0.5	24	15	1.2	13	0.5	4	4.5	1.4	12.5
Sp	1 700	51	3 200	14	110	772.8	51.2	196.8	1.5	30.9	33.4	4.3	95.9
YF	940	4.7	N/A	3.2	34	210.6	13.2	N/A	0.1	18.2	18.5	N/A	29.2
Total	14 000	350	8 300	56	700	3 593.1	352.1	728.8	7.7	239	251.4	15.8	603.4

DALY: disability-adjusted life-year; HepB: hepatitis B; Hib: *Haemophilus influenzae* type b; HPV: human papillomavirus; JE: Japanese encephalitis; N/A: not applicable; NmA: *Neisseria meningitidis* serogroup A; Sp: *Streptococcus pneumoniae*; US$: United States dollars; YF: yellow fever.

^a^ Estimates rounded.

^b^ Expressed in 2010 values.

^c^ Includes lifelong disabilities following childhood infection with Hib, JE, measles, NmA, rubella or Sp (Table 1).

By 2020, immunizations since 2001 will have averted an estimated US$ 350 billion (uncertainty range: 260–460 billion) in total costs due to illness. Most of these costs – about US$ 250 billion (uncertainty range: 190–330 billion) – will have been averted since 2011, of which about US$ 240 billion (uncertainty range: 180–320 billion) represents averted productivity loss caused by premature death. Between 2011 and 2020, US$ 4 billion (uncertainty range: 3–4 billion) in treatment costs, US$ 350 million (uncertainty range: 240–490 million) in transportation costs and US$ 730 million (uncertainty range: 650–790 million) in caregiver productivity losses could be averted. When we estimated the total cost of illness averted by vaccination against each of our study diseases, it appeared that greater costs could be averted, per vaccinated individual, through protection against *H. influenzae* type b, *S. pneumoniae*, human papillomavirus and measles than protection against any of the other six study diseases (Table 3).

**Table 3:** Costs of illness averted as the result of vaccinations against 10 diseases, 73 Gavi-supported low- and middle-income countries, 2001–2020

Antigen	Averted costs of illness (2010 US$)		
	Per vaccinated individual^a^	Per care-seeking case averted^b^	Per death averted^c^
Hepatitis B	61	12	7 000
*Haemophilus influenzae* type b	105	48	22 000
Human papillomavirus	102	7	4 000
Japanese encephalitis	9	200	35 000
Measles	85	7	27 000
*Neisseria meningitidis* serogroup A	25	31	17 000
Rotavirus	37	9	23 000
Rubella	5	71	16 000
*Streptococcus pneumoniae*	122	38	19 000
Yellow fever	72	10	19 000

US$: United States dollars.

^a^ The averted costs are both short-term – i.e. those incurred immediately at disease onset as the result of treatment, transportation or lost caregiver wages – and long-term – i.e. those associated with productivity lost, as a result of disease and/or disability, over the lifetime of the affected individual. This was calculated by dividing the total cost of illness by the number of individuals who received the recommended course of vaccine doses against that antigen.

^b^ These estimates are based only on averted short-term costs of illness. This estimates the averted cost of treatment, transportation and lost caretaker wages divided by the number of individuals with each vaccine-preventable disease who likely sought health-care treatment.

^c^ These estimates are based on data from care-seeking cases who subsequently died and exclude individuals with productivity loss due to disability.

**Fig. 1:**
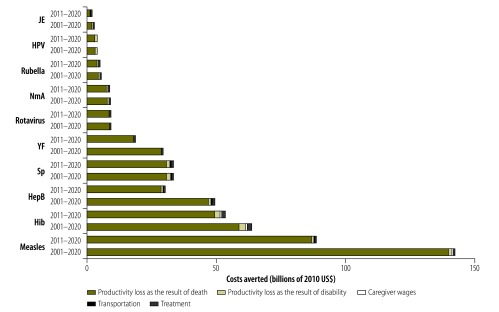
Costs of illness averted as the result of vaccinations against 10 diseases, 73 Gavi-supported low- and middle-income countries, 2001–2020 and 2011–2020

HepB: hepatitis B; Hib: *Haemophilus influenzae* type b; HPV: human papillomavirus; JE: Japanese encephalitis; NmA: *Neisseria meningitidis* serogroup A; Sp: *Streptococcus pneumoniae*; US$: United States dollars; YF: yellow fever.

Projected introductions of new vaccines and supplementary immunization activities account for US$ 170 billion – about 66% – of the estimated economic benefits of the Decade of Vaccines, with the remainder attributable to the scale-up in coverage of vaccines introduced before 2011. Supplementary immunization activities against measles represented the largest contributor to our estimates of the overall averted costs of illness – representing approximately US$ 130 billion (uncertainty range: 70–220 billion), or about 37% of total averted costs, and US$ 76 billion (uncertainty range: 42–133 billion), or about 30% of total averted costs, for the periods 2001–2020 and 2011–2020, respectively. The second, third and fourth largest drivers of the cost of illness appeared to be *H. influenzae* type b, *S. pneumoniae* and hepatitis B, respectively. Fig. 1 shows the disease-specific costs of illness averted by vaccination, for the periods 2001–2020 and 2011–2020. Most of these averted costs were represented by the productivity saved as the result of reduced mortality. Of the 73 countries included in the analysis, the five with the largest birth cohorts – i.e. Bangladesh, India, Indonesia, Nigeria and Pakistan – together accounted for more than half of the estimated total cost of illness averted between 2011 and 2020 (available from the corresponding author). In terms of the estimated total averted costs of illness per vaccinated individual (available from the corresponding author), the study countries in the WHO African Region came highest, at US$ 71, followed by those in the South-East Asia (US$ 58), Western Pacific (US$ 56), Eastern Mediterranean (US$ 45) and European (US$ 33) Regions and then the Region of the Americas (US$ 20).

Sensitivity analysis indicated that our estimates of the averted costs of illness were most sensitive to variation in estimates of the numbers of deaths averted by vaccination, particularly against diseases associated with a high disease burden and young age at onset – e.g. *H. influenzae* type b, measles, rotavirus and *S. pneumoniae* (available from the corresponding author). Cost parameters such as per-capita GDP proved to be less influential.

### Economic and social value

**Fig. 2:**
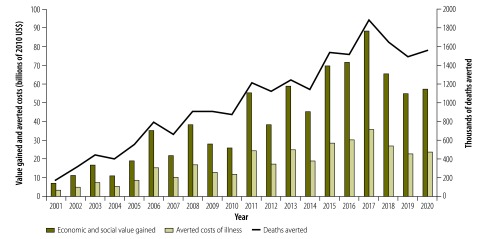
Economic and social value gained, averted costs of illness and deaths averted annually, as the result of vaccinations against 10 diseases, 73 Gavi-supported low- and middle-income countries, 2001–2020

In terms of their overall broader economic and social impact, we estimated the vaccinations we investigated to be worth approximately US$ 820 billion (uncertainty range: 560–1200 billion) and US$ 600 billion (uncertainty range: 420–870 billion) over the periods 2001–2020 and 2011–2020, respectively. About 97% of each of these values was represented by the value of averted mortality. Averted morbidity contributed much less – about US$ 19 billion (uncertainty range: 16–22 billion) and US$ 16 billion (uncertainty range: 14–19 billion) over the periods 2001–2020 and 2011–2020, respectively. Over half of the estimated economic and social value of vaccination in 2001–2020 is attributable to vaccinations against *H. influenzae* type b, hepatitis B and *S. pneumoniae*. Fig. 2 presents a year-on-year comparison of the estimated economic and social values, costs of illness averted and deaths averted between 2001 and 2020. The annual fluctuations shown are largely a result of supplementary immunization activities against measles, which are scheduled to peak every other year.

US$: United States dollars.

Similar to our estimates of the averted costs of illness, our broader estimates of the economic and social value of vaccinations were most sensitive to variation in the estimated health impact of vaccinations against childhood illnesses with a high disease burden – e.g. *H. influenzae* type b, measles, rotavirus and *S. pneumoniae*.

## Discussion

Between 2001 and 2020, according to our estimates, immunization against 10 vaccine-preventable diseases in 73 low- or middle-income countries will avert almost 20 million child deaths and save US$ 350 billion in costs of illness. More than two-thirds of these benefits are expected to accrue from new vaccine introductions and increases in immunization coverage during the Decade of Vaccines. Between 2001 and 2020 – just as a result of the vaccinations we investigated – each of our Gavi-supported study countries could expect to avoid a mean of approximately US$ 5 million in treatment costs per year.

Most of the economic benefits of the vaccines we investigated come – or are expected to come –from the long-term gains associated with a more productive workforce. Our examination of the broader economic and social value of such vaccines, beyond labour productivity, illustrates the substantial gains associated with vaccination, with the value for all 73 study countries estimated to reach US$ 820 billion over the 20 years since Gavi was launched in 2001. Unlike the lower estimates of the averted costs of treatment, our estimates of the broader economic and social value of vaccines reflect the non-economic value that people place on living longer and healthier lives.^25^^,^^33^ Sensitivity analyses indicate that future economic analyses on this topic could be made stronger by the collection of additional empirical data on disease burden.

Our main findings are similar to those of previous analyses of the health and economic impact of vaccinations. Using newer inputs for the health impact models developed in 2013,^6^ our estimates are based on updated data on immunization coverages and disease burden and a newer version of Gavi’s strategic demand forecast.^7^ We also excluded the impact of a routine first-dose of measles vaccination and used a different model to estimate the health impact of yellow fever vaccination. Whenever several different estimates of health impact were available from multiple models – as was the case for *H. influenzae* type b, human papillomavirus, rotavirus and *S. pneumoniae* – we incorporated ranges for the impact in our sensitivity analyses. A previous estimate of the number of deaths expected to be averted between 2011 and 2020 in our study countries as the result of vaccinations against the same 10 diseases – i.e. 13 million^6^ – is similar to our estimate, of 14 million, taking into account the various updates that we made across models.

While the method we followed to estimate the averted costs of illness was similar to that used in previous analyses,^34^ our analyses included an extension, upgrading and/or improvement of the diseases covered, health impact inputs, vaccine demand forecasts and data sources. The method we followed to produce our broader estimates of the economic and social value of vaccinations improves on earlier research^35^ by using the annual per-capita value of an increase in life expectancy and also by capturing the value of life lived in disability. Our estimates of annual treatment costs for specific diseases are similar to those of previous related cost–effectiveness studies. For example, the annual treatment and societal costs averted due to introduction of rotavirus vaccine in low- and middle-income countries were estimated to total US$ 440 million^36^ – when expressed in 2010 US$ – compared with our corresponding estimate of US$ 690 million.

Our analysis had several limitations. Because of a lack of relevant input data across countries and years, many health impact models are static, have limited country-level empirical data for some inputs and do not include long-term effects such as herd immunity. Given the current downward trend in child mortality and in the proportion of childhood deaths attributable to vaccine-preventable diseases, estimates of the projected, future, health and economic impact of vaccinations may be overestimates. Furthermore, work is currently underway to refine and improve health impact models by the inclusion of probabilistic uncertainty analysis and programmatic constraints such as delayed vaccination, partial dosing and relative coverage – i.e. the extent to which deaths may be clustered in unvaccinated groups. Our analysis did not include health-system contributions or any other costs of vaccination programmes.^37^

Our results are likely to have been influenced by the underlying disease burden, the duration of time between vaccination and the vaccine-preventable disease, the size of the eligible birth cohorts, immunization coverage rates and the effectiveness of vaccination programmes. In general, vaccination programmes based on highly efficacious vaccines that are given in early childhood and target pathogens causing acute disease would be expected to have relatively high economic benefits. Vaccines administered later in life that target chronic infections occurring at older ages – e.g. vaccines against human papillomavirus – would be expected to have less economic benefit. Data from the vaccination programmes of countries with large populations, high disease burdens and considerable economic output contributed disproportionately to our global estimates. We did not estimate treatment costs for long-term disability because there were no relevant data for many of our study countries. We also did not capture the impact made by the vaccines included in the original Expanded Programme on Immunization – e.g. the bacille Calmette–Guérin, diphtheria–tetanus–pertussis and polio vaccines and first doses of measles vaccine. In estimating the economic impact, we did not capture macroeconomic benefits – e.g. growth in gross domestic product – or the economic implications of demographic changes resulting from vaccination.^38^^–^^40^ In addition, empirical data on the value of a life-year are not available from interventions targeting children in low- and middle-income countries.

Despite these limitations, our results should give global decision-makers some idea of the full economic and social benefits that could be gained by increasing investments in immunizations. They have already informed Gavi’s investment strategy for the period 2016–2020 and highlighted the need for better global-level estimates of the economic impact of vaccination.^41^ Unlike the conservative estimates used in Gavi’s strategy, which incorporated additional uncertainty in the base parameters, our results were based on available coverage estimates and model outputs. It seems clear that, in averting substantial costs and potentially increasing economic productivity among the world’s poorest countries, the impact of immunization goes well beyond health.
